# Patterns of Emotional Eating Changes After Sleeve Gastrectomy

**DOI:** 10.1007/s11695-026-08847-3

**Published:** 2026-07-23

**Authors:** Caroline A. Stiver, A. Janet Tomiyama, Zachary N. Weitzner, Hila Zelicha, Edward H. Livingston, Yijun Chen

**Affiliations:** 1 Department of Psychology, University of California, Los Angeles, Los Angeles, CA USA; 2 David Geffen School of Medicine, University of California, Los Angeles, CA USA

**Keywords:** Metabolic and bariatric surgery, Sleeve gastrectomy, Emotional eating

## Abstract

**Background:**

Emotional eating is very common among metabolic and bariatric surgery (MBS) candidates. However, the changes of emotional eating after sleeve gastrectomy (SG), its impact on short-term weight loss outcomes, and sex differences in emotional eating are still not well established.

**Objective:**

To study the changes of emotional eating changes after SG and its impact on short-term weight loss.

**Setting:**

University Hospital, United States.

**Methods:**

122 patients who had SG were included. Patient demographics, weight loss, and pre- and post-operative emotional eating scores were collected. Emotional eating was measured by the Coping subscale of the Palatable Eating Motives Scale.

**Results:**

Of the 122 patients enrolled, 95 completed both the pre-operative and post-operative surveys (77.87%). For the whole study population, the average emotional eating scores decreased significantly from 1.31 ± 1.17 in pre-op to 1.01 ± 1.16 one year after SG (*p* = .007). Women reported greater emotional eating at baseline and had significantly lower scores after MBS, 1.44 ± 1.17 versus 1.05 ± 1.13 (*p* = .006). On the other hand, men had much lower emotional eating at baseline and had no changes after MBS, 0.90 ± 1.10 versus 0.86 ± 1.27, (*p* = .820). Greater emotional eating at baseline predicted greater excess body weight loss one year after surgery (*p* = .047). Although not statistically significant, there was a sharp increase of intense eating behavior after SG.

**Conclusion:**

Emotional eating decreased significantly in female patients, but not in male patients, one year after SG. Patients with higher emotional eating scores before surgery had greater weight loss one year after SG. MBS-induced weight loss may be mediated, in part, by improvements in eating behaviors.

**Supplementary Information:**

The online version contains supplementary material available at https://doi.org/10.1007/s11695-026-08847-3.

## Key Points

One year after bariatric surgery, emotional eating levels significantly decreased in female patients.

Higher emotional eating at baseline predicted greater weight loss one year after surgery.

**Supplementary Information:**

The online version contains supplementary material available at https://doi.org/10.1007/s11695-026-08847-3.

## Introduction

Metabolic and bariatric surgery (MBS) has consistently been shown to be an efficacious and long-lasting treatment for weight loss in individuals with obesity [1–3]. Although the wide-ranging benefits of bariatric surgery have been well established, procedural results often vary from person-to-person. This variation underscores a need to better understand which factors contribute to individual differences in postsurgical outcomes and identify ways to predict those who will fail to lose adequate weight or have significant weight regain after surgery.

One such factor is emotional eating. Emotional eating, an eating behavior elicited by emotional triggers (particularly negative emotions such as fear, anger, and sadness), has been associated with obesity and is very common among people who undergo MBS [4–6]. Emotional eating may affect weight loss outcomes, making it a potentially important factor to consider in optimizing MBS-related weight loss. Emotional eating changes after MBS have been reported by many groups in the past, most in patients who had Roux-en-Y gastric bypass surgery. A meta-analysis of 23 studies revealed that the emotional eating score decreased 12 months after MBS and the results were mixed after 12 months [7]. The vast majority of the participants included in the meta-analysis had Roux-en-Y gastric bypass surgery. Only a few studies with a small number of patients have investigated emotional eating after sleeve gastrectomy (SG). In a cross-sectional study of 300 Pakistani adults, gastric bypass surgery patients exhibited higher emotional eating compared to SG patients [8]. Wang et al. reported improved self-reported emotion-related eating behavior 12 months after SG in 30 Chinese patients with obesity [9]. On the other hand, “emotional eating behavior” scores declined only temporarily in the early post-operative period of one month, but returned to the pre-operative level at 12 months in 49 Japanese patients [10]. The small number of patients and inconsistent data suggest a need for larger studies on emotional eating after SG. Larger studies offer greater precision and statistical power, leading to more reliable and robust findings.

Compared with men, emotional eating is more common in women, and women are also more likely to experience mood-related disorders and eating disorders [11, 12]. While men and women have similar obesity prevalence, a substantial gender disparity has persisted over the past few decades, with men comprising only 20% of patients undergoing MBS. There are also differences in MBS outcomes between men and women. A large study with more than 107,504 participants showed that male patients lose slightly more weight, but had slightly higher incidence of complications [13].

Although there were many studies looking at emotional eating after MBS, no studies have examined sex differences in emotional eating change before and after MBS. Understanding emotional triggers for eating in men and women before and after SG may help us to develop tailored interventions for people seeking obesity treatment, of whom a large majority are women.

Although MBS leads to sustainable weight loss, significantly improved comorbidity, and improved psychological well-being, weight regain following MBS has been linked to elevated risks of alcohol and substance abuse, suicidal ideation and suicide attempts, and accidental death [14]. It is not well understood why intense psychological behaviors increase, while general psychological status improves after MBS. Intense emotional eating behavior includes almost always/always eating palatable food to forget about problems or worries. To our knowledge, no studies have ever investigated intense emotional eating behavior after MBS.

We hypothesized that emotional eating would improve after SG, similar to the pattern for Roux-en-Y gastric bypass surgery. We also hypothesized that there would be a gender difference in emotional eating before and after surgery. In this study, therefore, we aimed to investigate (1) how emotional eating patterns change after SG, (2) the sex differences in these changes, (3) pre-operative level of emotional eating as a predictor of weight loss outcomes, and (4) the changes of intense eating behavior after SG.

## Methods

This study was a longitudinal cohort study designed to investigate the role of emotional eating in the context of bariatric surgery. All methods were approved by the UCLA Institutional Review Board (#14–000486).

### Participants

Patients who underwent SG at the Ronald Reagan UCLA Medical Center between January 1, 2019 and February 29, 2024 were invited to participate in the study. Those undergoing revisional or endoscopic bariatric procedures were excluded. After providing informed consent, participants (*N* = 122) completed the measures through a digital questionnaire administered on the Qualtrics platform (Provo, UT, USA) at their pre-operative appointment (usually 6 weeks before surgery). At their one-year follow-up appointment, they were asked again to complete the follow-up survey using the identical platform (*N* = 95; 77.87% retention rate).

### Measures

Emotional eating was measured by the Coping subscale of the Palatable Eating Motives Scale, a 4-item Likert scale with 5-point response options ranging from “Almost never/Never” to “Almost always/Always” [15]. Participants were given examples of palatable foods (e.g., sweets, salty snacks, fast foods, and sugary drinks) and asked how often they ate these foods for reasons such as “To forget your worries,” and “To cheer up when you’re in a bad mood.” Items were averaged to create a mean score; higher scores indicated greater tendency to consume palatable foods in response to negative emotions. The internal consistency of this measure was excellent (baseline Cohen’s α = 0.92; one-year Cohen’s α = 0.94). Excess body weight loss (EBWL) and total weight loss (TWL) were confirmed via electronic medical records and UCLA’s Metabolic and Bariatric Surgery Accreditation and Quality Improvement Program database. Sociodemographic information was self-reported.

### Analytic Plan

The analytic sample comprised those who completed both the baseline and follow-up surveys. Descriptive statistics were calculated, and paired-sample *t-*tests and analysis of variance (ANOVA) analyses examined differences pre- and post-operatively for emotional eating for sex and race/ethnicity^1^, respectively. Regression analyses tested the hypothesis that emotional eating at baseline would predict excess body weight loss. Paired-sample *t*-tests tested whether emotional eating scores one year post-surgery were lower than baseline scores. Repeated measures ANOVA models tested modification by sex in separate models. All α levels were set to a *p*-value of 0.05.

## Results

During the study period, a total of 122 patients completed the pre-operative survey prior to SG. Of these 122 patients, 95 completed both the pre-operative and post-operative surveys for a retention rate of 77.87%. Demographic data of the study group (those who completed both pre- and post-op surveys) are included in Table 1. Our study group is consistent with most bariatric surgery populations in the U.S., with 76.8% of the participants being female. 

**Table 1:** Demographics of the study sample

	*N* = 95
Age [mean (SD)]	42.60 (11.56)
Female [*n* (%)]	73 (76.8%)
Race/ethnicity [*n* (%)]	
White	39 (41.1%)
Hispanic or Latine	23 (24.2%)
Black or African American	14 (14.7%)
More than one race	7 (7.4%)
Asian	5 (5.3%)
Other	5 (5.3%)
Native Hawaiian	1 (1.1%)
Education [*n* (%)]	
Bachelor’s degree	39 (41.4%)
Graduate or Professional degree	22 (23.2%)
Some college without a college degree	14 (14.7%)
Associate’s degree	11 (11.65)
High school diploma, GED, or equivalent	9 (9.5%)
Annual family income before tax [mean (*SD*)]	$137,699 ($96,495)
Pre-operative Body Mass Index (kg/m^2^) [mean (*SD*)]	44.93 (7.53)
Post-operative Body Mass Index (kg/m^2^) [mean (*SD*)]	32.22 (5.81)
Pre-operative Weight (kg) [mean (*SD*)]	125.73 (25.18)
Post-operative Weight (kg) [mean (*SD*)]	89.84 (19.17)
Excess Body Weight Loss (%) [mean (*SD*)]	67.58 (22.13)
Total Body Weight Loss (%) [mean (*SD*)]	28.21 (8.39)

**Fig. 1:**
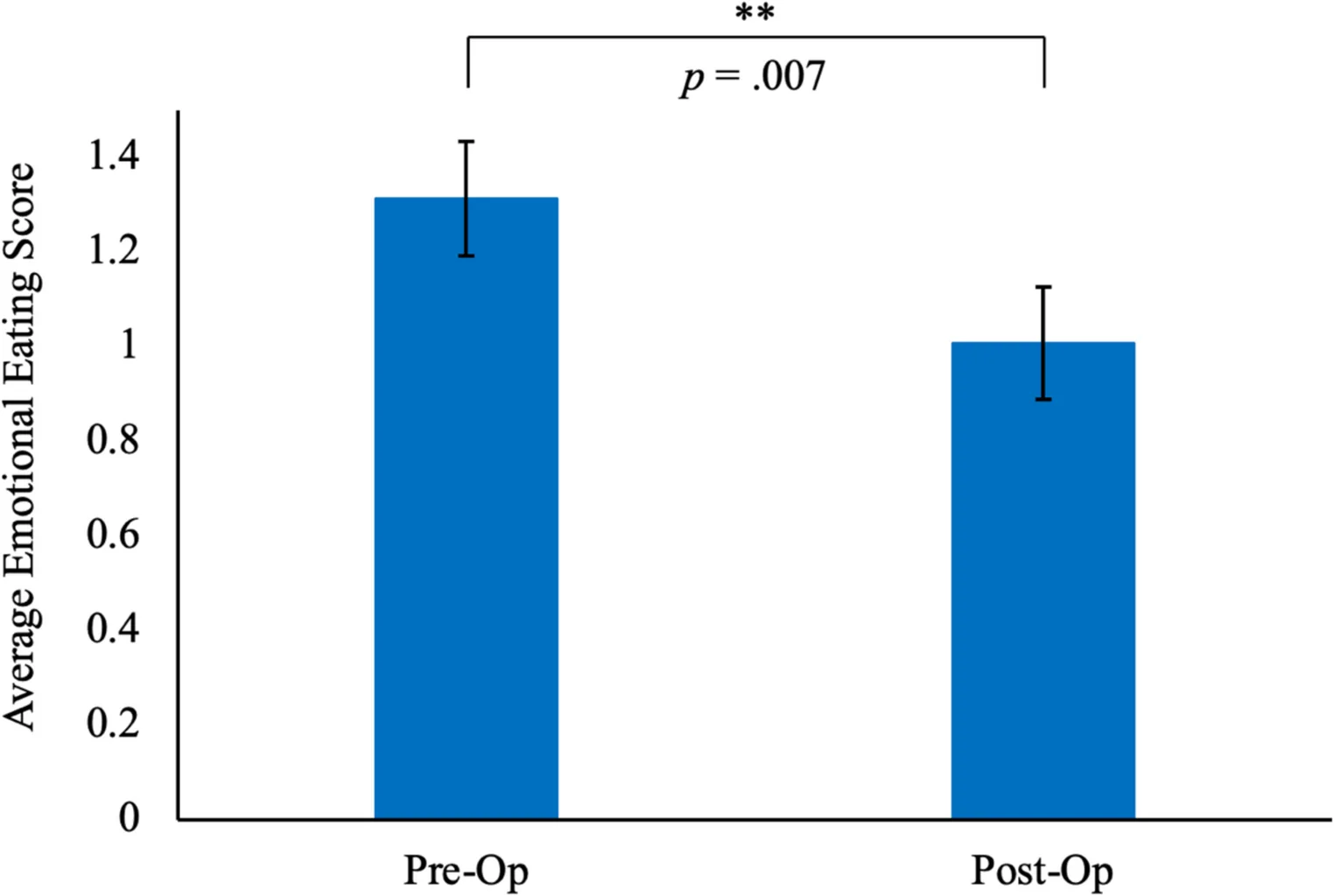
Change in emotional eating before and after sleeve gastrectomy

**Fig. 2:**
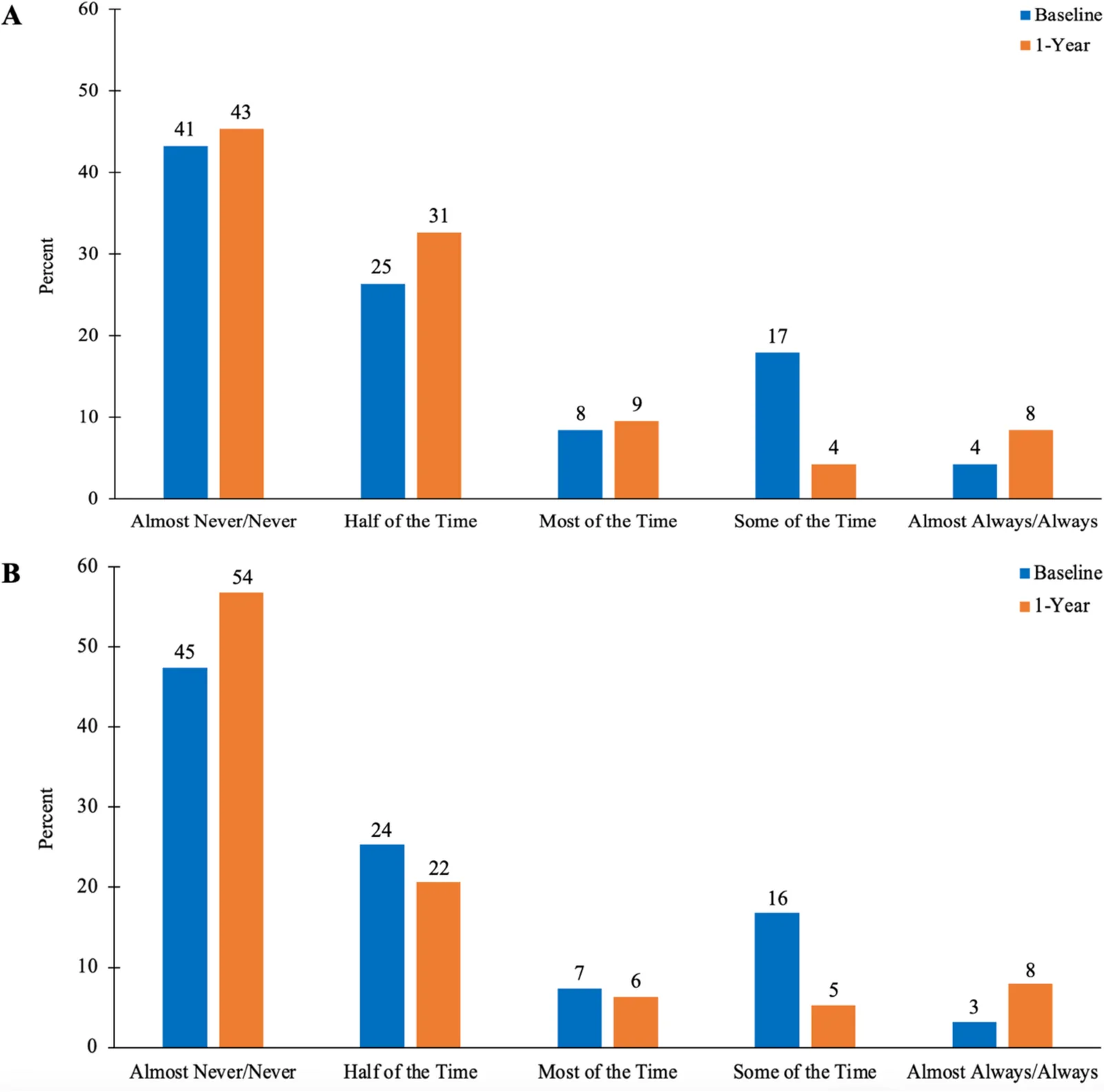
Intense eating behavior before and after sleeve gastrectomy. Note. Participants answered how often they ate palatable foods to forget their worries (**A**) and to forget about their problems (**B**). Raw numbers of patients are included on top of each data bar

Although overall emotional eating scores decreased significantly (Fig. 1), and most patients had less emotional eating behavior after surgery, we observed a sharp increase in a subset of those who responded “Almost always/Always” to eating “To forget your worries” (from 4.21% who endorsed “Almost always/Always” before surgery to 8.42% who endorsed “Almost always/Always” after surgery) and “To forget about your problems” (from 3.15% who endorsed “Almost always/Always” before surgery to 8.42% who endorsed “Almost always/Always” after surgery; Fig. 2). McNemar tests assessed whether the proportion of patients with intense eating pattern responses changed from pre- to post-surgery. Individual items of the Coping subscale of the Palatable Eating Motives Scale were dichotomized such that responses of “Almost always/Always” received a score of 1 and all other responses received a score of 0. Results showed no significant change in eating to forget worries [χ^2^ (1) = 1.125, *p* = .289], help when feeling depressed or nervous [χ^2^ (1) = 0.000, *p* = 1.000], cheer up [χ^2^ (1) = 0.364, *p* = .547], or forget problems [χ^2^ (1) = 2.286, *p* = .131]. Although this sharp increase was not statistically significant due to the small number of patients exhibiting this intense behavior, this finding is notable given that overall emotional eating improved significantly in our study population.

As expected, women had a higher baseline emotional eating score before MBS versus men: 1.44 ± 1.17 versus 0.90 ± 1.10 [*t*(93) = 1.924, *p* = .029] (Table 2). One year after surgery, there was a significant decrease of emotional eating score in women [1.44 ± 1.17 to 1.05 ± 1.13, *t*(72) = 2.843, *p* = .006]. Interestingly, there was little change in emotional eating score in men (0.90 ± 1.10 to 0.86 ± 1.27, *t*(21) = 0.230, *p* = .820). With this change, the emotional eating score was similar between women and men one year after SG [*t*(93) = 0.664, *p* = .508). A two-way repeated measures ANOVA testing for sex differences across time revealed no significant difference in emotional eating scores [*F*(1,93) = 2.592, *p* = .111], even after accounting for sex [*F*(1,93) = 1.821, *p* = .181]. Similarly, men and women displayed similar changes in emotional eating in the year following SG [*F*(1, 93) = 2.126, *p* = .148]. Results of sex difference analyses are provided in Tables 3, 4 and 5.

**Table 2:** Key study variables by sex

	Sex		*p*	Full sample
	Female	Male		
Pre-operative emotional eating (*N* = 95) [mean (*SD*)]	1.44 (1.17)	0.90 (1.10)	0.029*	1.31 (1.17)
1-year emotional eating (*N* = 95) [mean (*SD*)]	1.05 (1.13)	0.86 (1.27)	0.508	1.01 (1.16)
Excess body weight loss (% (*N* = 95)) [mean (*SD*)]	69.39 (22.42)	60.95 (20.19)	0.131	67.58 (22.13)
Total body weight loss (% (*N* = 95)) [mean (*SD*)]	28.47 (7.99)	27.27 (9.90)	0.572	28.21 (8.39)

Note. The *p*-value for the comparison of pre-operative emotional eating scores between female and male is one-sided and significant at the *p* < .05 level

**Table 3:** Results for independent samples *t*-tests

Time				95% Confidence interval		
	t	df	*p* (two-sided)	Lower	Upper	Cohen’s d
Pre-operative emotional eating	1.924	93	0.029*	− 0.015	0.948	0.468
1-year emotional eating	0.664	93	0.508	− 0.316	0.638	0.161

Note. The *p*-value for the comparison of pre-operative emotional eating scores between female and male is one-sided and significant at the *p* < .05 level

**Table 4:** Results for paired samples *t*-tests

Sex				95% Confidence interval		
	t	df	*p* (two-sided)	Lower	Upper	Cohen’s *d*
Female (*n* = 73)	2.843	72	0.006**	0.096	0.567	0.333
Male (*n* = 22)	0.230	21	0.820	− 0.370	0.467	0.049

Note. The *p*-value for the comparison of pre-operative and post-operative emotional eating scores for females is significant at the *p* < .01 level

**Table 5:** Results for two-way repeated measures ANOVA

	SS	df	MS	F	*p*	𝜂^2^_*p*_
Within-subjects (Time)						
Emotional eating	1.499	1	1.499	2.592	0.111	0.027
Emotional eating x Sex	1.053	1	1.053	1.821	0.181	0.019
Error	53.771	93	0.578			
Between-subjects (Sex)						
Sex	4.484	1	4.484	2.126	0.148	0.022
Error	196.120	93	2.109			

Note. SS represents Type III Sum of Squares; MS refers to Mean Square

**Fig. 3:**
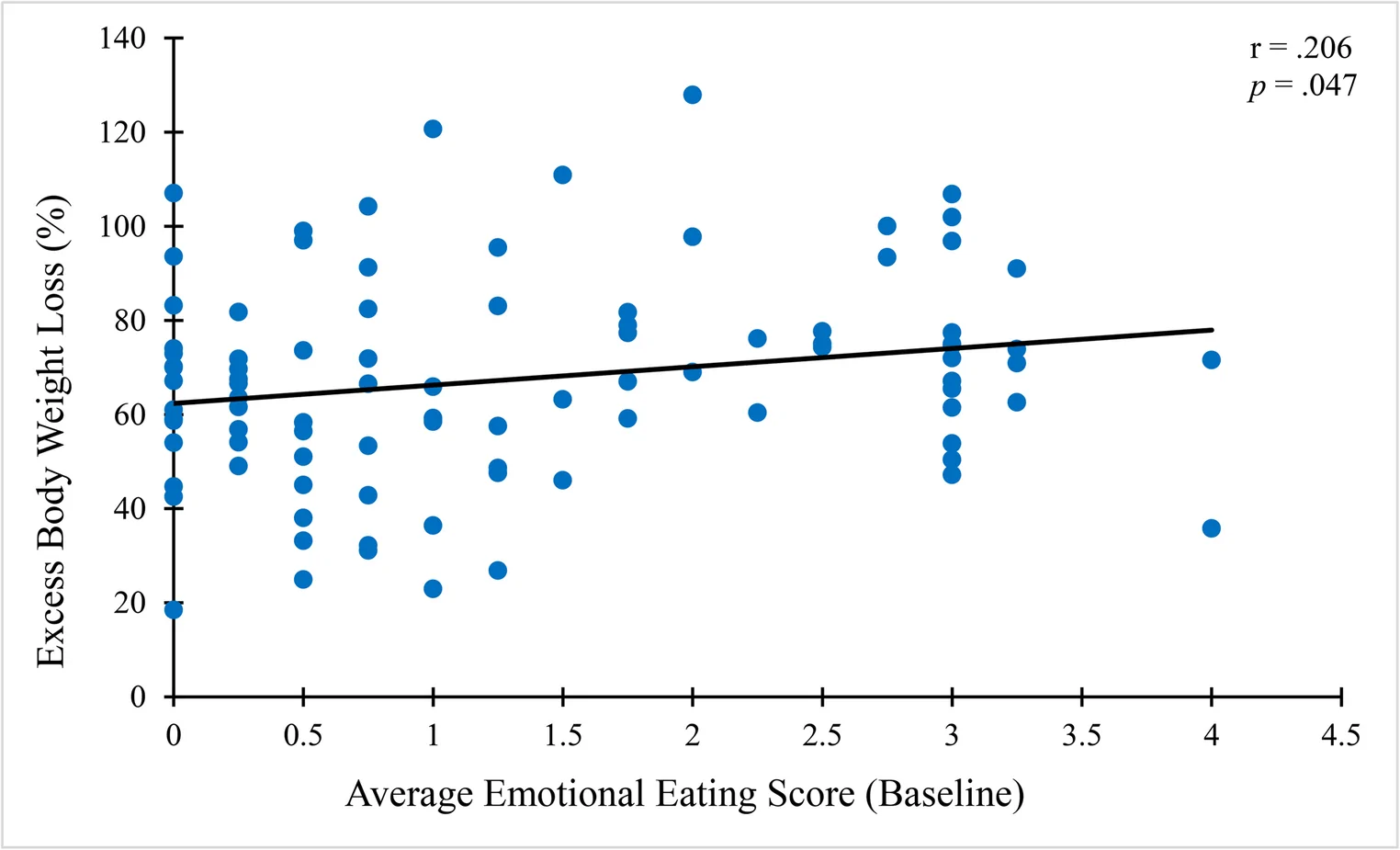
Pre-operative emotional eating and excess body weight loss after sleeve gastrectomy

For the whole study cohort, the average excess body weight loss was 67.6% and women had higher EBWL% than men (69.4% versus 60.9%,). *The average total weight loss for the complete sample was 28.2%; women showed marginally higher TWL% than men (28.5% versus 27.3%).* Higher baseline emotional eating predicted *better* weight loss one year after surgery, *(p* = .047) (Fig. 3). The data suggest that emotional eating may have contributed to weight gain before surgery and the correction of this pathological eating behavior helped improve weight loss after SG.

## Discussion

In this study, we showed that emotional eating declined overall post-SG, suggesting salubrious effects of bariatric surgery on this eating pattern in most patients. These data are similar to prior reports showing emotional eating improved after Roux-en-Y gastric bypass surgery [7]. Our study is the largest study to date to investigate emotional eating after SG. Together with the prior literature, we believe that both Roux-en-Y gastric bypass and SG can reduce emotional eating at one year after surgery. The smaller stomach size that limits food intake, and hormonal changes that decrease hunger and increase fullness might be the main mechanisms of this reduced emotional eating after SG. Patients may not get the same satisfaction, relief of stress/worries while eating palatable foods after SG, thus reducing the emotional eating. Even though the emotional eating measure utilized references eating highly palatable food in response to emotions, we would assume that patients should have a similar reduction of emotional eating after SG towards non-highly palatable foods. We also theorize that this new healthier eating habit contributed to weight loss. Patients with higher emotional eating scores had greater weight loss, suggesting that emotional eating drove patients’ weight gain before surgery. Prior work on dysfunctional eating behaviors in the context of eating disorders revealed emotional eating patterns vary across weight status and disorder type. Individuals who were overweight or had bulimia nervosa were found to consume more food in response to negative emotions than those who were underweight or had anorexia nervosa, who ate less than individuals of normal weight [16]. Such findings suggest that emotional eating may function as a pathological behavior associated with disordered eating. As such, once this pathological eating behavior was corrected, patients may have lost the weight gain caused by emotional eating. This is consistent with the pattern we observed in our study wherein emotional eaters lost more weight from SG than those with lower levels of emotional eating. Our data also suggest that high level of emotional eating before MBS should not be considered a major psychological concern because it will likely improve after surgery.

In addition to this significant difference in the utilization of MBS between male and female patients, other sex differences in MBS outcomes have been reported [13]. To our knowledge, this is the first study to show sex differences in emotional eating after MBS. Emotional eating only decreased in female patients one year after MBS, while there was no change in male patients. Consistent with previous findings, our study revealed that female patients reported significantly greater levels of emotional eating pre-surgery than male patients [11, 12]. In the general population, eating is frequently used as a means of comfort to manage depressed mood or negative emotions. Mood disorders, anxiety, and depression are much more prevalent in women than men [11, 12]. It is well established that MBS decreases anxiety and improves depression. Therefore, it makes sense that we observed improved emotional eating scores for female patients after SG in our study. However, we were still surprised to see that the emotional eating score did not change at all for male patients one year after SG. Based on the data, we speculate that the emotional eating is one of the major contributors for weight gain in female patients with obesity, while it may not be a major contributor of weight gain in men. Our study adds to the literature a unique aspect of sex differences in MBS: the divergence of emotional eating change after MBS. With overall improvement of emotional eating after MBS, however, we noted that the raw number of those reporting intense emotional eating behavior doubled one year after SG. For instance, the number of patients who reported eating to forget worries increased from 4 to 8, while those who endorsed eating to solve problems increased from 3 to 8. Although the absolute number of this subgroup of patients was small, these findings were unexpected. These findings mirror those observed in the context of depression and suicide post-bariatric surgery. Although MBS leads to a significant improvement of depression in most patients, suicidal and self-harm risks go up in a small number of individuals after MBS [14]. Therefore, assessing emotional eating from baseline throughout all follow-up visits may be warranted. A larger study is needed to study this intense eating pattern after MBS.

This study had several limitations. (1) The findings can only be generalized to those undergoing SG and not necessarily those undergoing alternative bariatric surgery procedures such as Roux-en-Y gastric bypass surgery or adjustable gastric banding. (2) Although this is among the largest studies of emotional eating in bariatric surgery, and the first to test the roles of sex to our knowledge, we nonetheless may have been underpowered to detect two-way interactions. (3) Even though our study is the largest to date to investigate the eating pattern after SG, we only had 95 patients in our cohort. While we saw a statistical difference using % EBWL, we did not see this difference using % TWL. (4) The results of our study may not be directly comparable to other studies because we used the Coping subscale of the Palatable Eating Motives Scale, while much of the literature on emotional eating and MBS utilized the Emotional Eating Scale [15]. (5) Finally, the effects we observed were small (about one third of a point on a 1–5 scale). Although the measure showed excellent reliability in our sample, there is potential for socially desirable responses among participants when completing items assessing emotional eating behaviors. Nevertheless, this is one of first studies to investigate whether changes in emotional eating patterns following bariatric surgery varied by sex. These findings address a critical gap in the existing literature and provide necessary information regarding how sociodemographic differences may impact patient outcomes.

## Conclusion

Emotional eating levels significantly decreased one year after SG, indicating a behavioral pathway through which MBS leads to weight loss. The decrease of emotional eating only occurred in female patients, not in male patients. Higher pre-operative emotional eating predicts greater weight loss one year after SG. Future research should further investigate rare but potentially serious increases in the intense emotional eating after MBS and its effect on weight loss and psychological wellbeing.

## Supplementary Information

Below is the link to the electronic supplementary material.

Supplementary Material 1 (DOCX 22.9 KB)

## Data Availability

No datasets were generated or analysed during the current study.

## Footnotes

1 We note that we also conducted analyses comparing emotional eating between race/ethnicity. All results were null, and these analyses are available in Supplementary Materials.
