# Supplementary material for: Patterns of Emotional Eating Changes After Sleeve Gastrectomy
Source: Obes Surg. 2026 Jul 23;36(9):4626–33. doi: 10.1007/s11695-026-08847-3 (PMC13562336; doi:10.1007/s11695-026-08847-3)
Supplement: Supplementary file 1 [file 11695_2026_8847_MOESM1_ESM.docx]

**Supplementary Materials**

**Examining racial/ethnic differences in patterns of emotional eating**

- **Analytic Plan:** Descriptive statistics were calculated and ANOVA analyses were run to examine racial/ethnic differences in pre- and post-operative emotional eating, pre- and post-operative body weight, and excess body weight loss. A two-way Repeated Measures Analysis of Variance model tested modification by race/ethnicity. Due to cell sizes, race was collapsed into three categories of White, Black, and Hispanic. All α levels were set to a p-value of .05
- **Results:** Emotional eating scores were not found to significantly differ across racial/ethnic groups at baseline (*p* = 0.572) or one year after surgery (*p* = 0.870).

**Results**

- **One-Way ANOVAs:** Results of multiple one-way ANOVAs showed no significant difference in pre- and postoperative emotional eating, pre and postoperative body weight, or EBWL across racial/ethnic groups.
- **Two-Way Repeated Measures ANOVA:** Results of the two-way repeated measures ANOVA assessing change in preoperative and postoperative emotional eating across racial/ethnic groups, showed a significant difference in emotional eating in the 1-year following SG [*F*(1,73) = 4.136, *p* = .046]. The interaction between emotional eating and race/ethnicity however, was not significant [*F*(2,73) = 1.810, *p* = .171]. Similarly, between-subjects effects revealed no significant difference in emotional eating across racial/ethnic groups [*F*(2,73) = 1.017, *p* = .367].

**Table 1** Descriptive statistics by race/ethnicity

|  | **White**  **(*n* = 29)** | | **Black/African American (*n* = 13)** | | **Hispanic/Latine**  **(*n* = 18)** | |
| --- | --- | --- | --- | --- | --- | --- |
|  | ***M*** | ***SD*** | ***M*** | ***SD*** | ***M*** | ***SD*** |
| Preoperative emotional eating | 1.388 | 1.139 | 1.269 | 1.166 | 1.000 | 1.377 |
| 1-year emotional eating | 1.078 | 1.144 | 0.885 | 0.933 | 1.097 | 1.478 |
| Preoperative body weight (kg) | 130.172 | 27.300 | 130.267 | 23.545 | 124.756 | 32.478 |
| 1-year body weight (kg) | 92.554 | 20.959 | 91.878 | 17.224 | 87.971 | 17.232 |
| Excess body weight loss (%) | 69.583 | 24.532 | 66.782 | 18.170 | 67.725 | 16.530 |

***Note.* Sample sizes reflect the number of patients in each racial/ethnic group following listwise deletion.**

**Table 2** Results of one-way ANOVA by race/ethnicity

|  |  |  |  |  |  | **95% Confidence Interval** | |  |
| --- | --- | --- | --- | --- | --- | --- | --- | --- |
|  | **SS** | ***df*** | ***MS*** | ***F*** | ***p*** | **Lower** | **Upper** | **𝜂*^2^*** |
| Preoperative emotional eating | 1.680 | 2 | .840 | .564 | .572 | .000 | .110 | .019 |
| 1-year emotional eating | .414 | 2 | .207 | .140 | .870 | .000 | .056 | .005 |
| Preoperative body weight (kg) | 373.798 | 2 | 186.899 | .234 | .792 | .000 | .074 | .008 |
| 1-year body weight (kg) | 245.123 | 2 | 122.561 | .334 | .717 | .000 | .087 | .012 |
| Excess body weight loss (%) | 82.803 | 2 | 41.401 | .093 | .912 | .000 | .042 | .033 |

***Note.* White (*n* = 29), Black/African American (*n* = 13), Hispanic/Latine (*n* = 18).**

**Table 3** Results for two-way repeated measures ANOVA

|  | **White (*n* = 39)** | **Black/African American (*n* = 14)** | **Hispanic/Latine (*n* = 23)** |
| --- | --- | --- | --- |
| Preoperative emotional eating [*M*, *SD*] | 1.519, 1.148 | 1.393, 1.212 | .902, 1.292 |
| 1-year emotional eating [*M*, *SD*] | 1.180, 1.210 | .893, .897 | .978, 1.365 |

|  | **SS** | ***df*** | ***MS*** | ***F*** | ***p*** | **𝜂*^2^_p_*** |
| --- | --- | --- | --- | --- | --- | --- |
| *Within-Subjects (Time)* |  |  |  |  |  |  |
| Emotional Eating | 2.075 | 1 | 2.075 | 4.136 | .046* | .054 |
| Emotional Eating x Race | 1.816 | 2 | .908 | 1.810 | .171 | .047 |
| Error | 36.620 | 73 | .502 |  |  |  |
| *Between-Subjects (Race)* |  |  |  |  |  |  |
| Race | 4.912 | 2 | 2.456 | 1.017 | .367 | .027 |
| Error | 176.307 | 73 | 2.415 |  |  |  |

***Note.* The p-value for the comparison of preoperative and postoperative emotional eating scores for is significant at the p < .05 level.**

**Conclusions**

- Results indicated that White, Black/African American, and Hispanic/Latine patients experienced similar changes in emotional eating across time.
- **Limitations:** Based on the demographics of the population available to us, we could not assess race/ethnicity interactions for Asian or Native Hawaiian participants and those reporting more than one race or other races.
